# Machine learning—aided personalized DTI tractographic planning for deep brain stimulation of the superolateral medial forebrain bundle using HAMLET

**DOI:** 10.1007/s00701-019-03947-9

**Published:** 2019-05-30

**Authors:** Volker A. Coenen, Thomas E. Schlaepfer, Peter C. Reinacher, Hansjoerg Mast, Horst Urbach, Marco Reisert

**Affiliations:** 1 Department of Stereotactic and Functional Neurosurgery, Neurocenter - University Medical Center, Breisacher Straße 64, 79106 Freiburg i.Br., Germany; 2 Medical Faculty, Freiburg University, Freiburg, Germany; 3 BrainLinks/BrainTools, Cluster of Excellence, Freiburg University, Freiburg, Germany; 4 Department of Interventional Biological Psychiatry, Freiburg, Germany; 5 Department of Neuroradiology, Freiburg University Medical Center, Freiburg, Germany

**Keywords:** Brain, Deep brain stimulation, Depression, Machine learning, Medial forebrain bundle, Obsessive-compulsive disorder, Stereotaxy

## Abstract

**Background:**

Growing interest exists for superolateral medial forebrain bundle (slMFB) deep brain stimulation (DBS) in psychiatric disorders. The surgical approach warrants tractographic rendition. Commercial stereotactic planning systems use deterministic tractography which suffers from inherent limitations, is dependent on manual interaction (ROI definition), and has to be regarded as subjective. We aimed to develop an objective but patient-specific tracking of the slMFB which at the same time allows the use of a commercial surgical planning system in the context of deep brain stimulation.

**Methods:**

The HAMLET (Hierarchical Harmonic Filters for Learning Tracts from Diffusion MRI) machine learning approach was introduced into the standardized workflow of slMFB DBS tractographic planning on the basis of patient-specific dMRI. Rendition of the slMFB with HAMLET serves as an objective comparison for the refinement of the deterministic tracking procedure. Our application focuses on the tractographic planning of DBS (*N* = 8) for major depression and OCD.

**Results:**

Previous results have shown that only fibers belonging to the ventral tegmental area to prefrontal/orbitofrontal axis should be targeted. With the proposed technique, the deterministic tracking approach, that serves as the surgical planning data, can be refined, over-sprouting fibers are eliminated, bundle thickness is reduced in the target region, and thereby probably a more accurate targeting is facilitated. The HAMLET-driven method is meant to achieve a more objective surgical fiber display of the slMFB with deterministic tractography.

**Conclusions:**

The approach allows overlying the results of patient-specific planning from two different approaches (manual deterministic and machine learning HAMLET). HAMLET shows the slMFB as a volume and thus serves as an objective tracking corridor. It helps to refine results from deterministic tracking in the surgical workspace without interfering with any part of the standard software solution. We have now included this workflow in our daily clinical experimental work on slMFB DBS for psychiatric indications.

## Introduction

There is a growing interest in deep brain stimulation (DBS) of the superolateral medial forebrain bundle (slMFB) for the alleviation of otherwise treatment-resistant psychiatric disorders like major depression (MD) [2, 3, 14, 15, 25] and obsessive-compulsive disorder (OCD) [11, 17]. The anatomical structure itself was only recently characterized [1, 9, 13]. The slMFB has been researched in a variety of psychiatric conditions [4, 5, 20, 28]. DBS of the slMFB is performed under tractographic assistance [10, 14, 25] and addresses fibers that project from the ventral tegmental area (VTA) to the prefrontal (PFC) and orbitofrontal (OFC) cortices. An anatomically plausible display of the structure in a surgical planning system is dependent on a variety of factors (e.g., MRI/DWI sequence quality, field strength, accuracy of ROI definition, algorithms used). The result is a streamline image of the slMFB that might be hard to appreciate for the untrained eye, especially because of its complex course through the brain, its far-reaching connection to the frontal lobe [1, 10], and its proximity to the anterior thalamic radiation [9]. Since connectome anatomy is displayed, a refinement of peripheral fiber segments (e.g., over-sprouting motor-related fibers) influences the definition of the target region [1] deeming the tractographic process as subjective. Despite the use of clearly defined ROIs and procedural definitions [1, 8–10], individual tractographic display of the slMFB remains subjective and is influenced—amongst others—by factors like quality of DTI MRI data, appreciation of the anatomical structure, and accuracy of the ROI definition. So far, only visual appreciation could be used to control for aberrant display of fibers that do not belong to the main structure. Especially over-sprouting of motor-related fibers makes a definition of the target region challenging [1]. It was shown that the fibers connecting VTA and PFC/OFC are needed for effective stimulation and treatment of psychiatric disorders [1, 10, 13, 14, 25]. Aberrant fibers which are additionally seen in the periphery will enlarge the thickness and will alter the position of the target bundle in the target region (Fig. 5) and thereby diminish targeting accuracy in the therapeutic triangle.

**Fig. 1:**
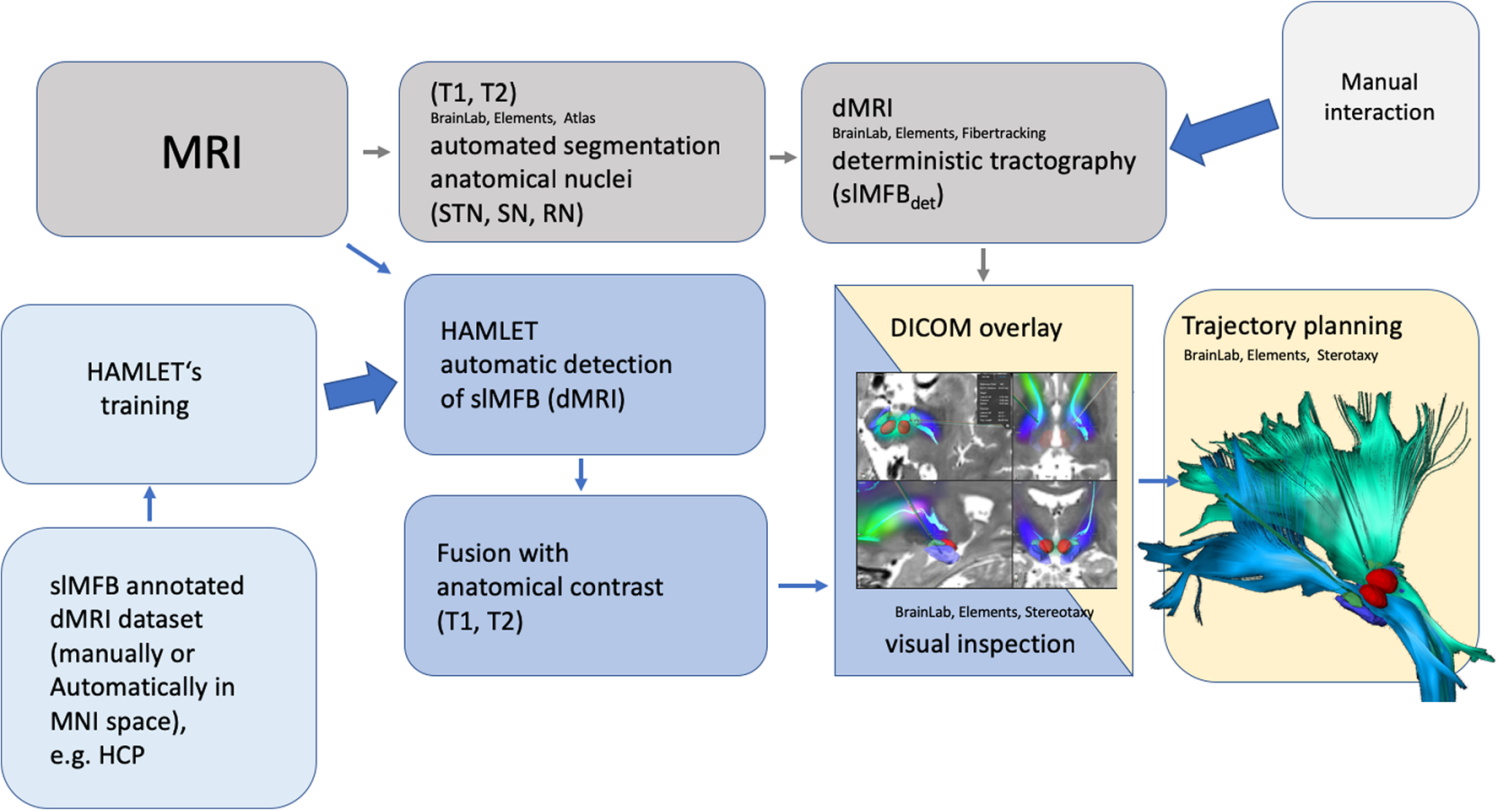
Workflow for the planning of slMFB DBS. Machine learning pipeline and depiction of the slMFB (blue background). Standard workflow of anatomical segmentation of nuclei (STN, SN, RN), deterministic tracking of slMFB and trajectory planning (gray background). Note: Although DICOM data is included with slMFBml overlay, the standard planning software is in no way affected. DICOM overlay serves as a comparison to check the plausibility of slMFBdet rendition only. Acceptance of result and end check is left to the discretion of the operating expert. Legend: STN, subthalamic nucleus; SN, substantia nigra; RN, red nucleus; det, deterministic tractography; ml, machine learning

We here introduce the clinical application of a machine learning process called HAMLET (Hierarchical Harmonic Filters for Learning Tracts from Diffusion MRI) (Fig. 1). HAMLET is a tract learning algorithm and is able to directly map raw diffusion MRI data onto a directional map indicating tract presence and direction. Training of HAMLET is accomplished on a set of healthy controls, which all underwent a conventional global tractography followed by a selection procedure for the tracts of interest. After training, it automatedly analyzes individual diffusion-weighted MRI (dMRI) sequences and maps the result as a color-coded image, which is overlaid on an ordinary anatomical contrast and written in an RGB image (DICOM—Digital Imaging and Communications in Medicine). In our case, it learns the slMFB structure and automatedly maps it onto the patient-specific images using the individual DWI sequences which are also used for deterministic tractography and trajectory planning [21].

Although it appears to be relevant to refine the individual deterministic tractographic approach of slMFB streamline rendition in order to allow a more objective tracking process, the process should seamlessly be integrated into the surgical planning process (thus it should stay in the same planning environment). Our goal was an objective and anatomically plausible but also personalized planning of deterministic slMFB streamlines, which will serve for surgical planning of DBS procedures.

## Material and methods

### Patients

Demographic data are listed in Table 1.

**Table 1:** Demographic data (Legend: OCD= obsessive compulsive disorder; TRD= treatment resistant depression. Baseline YBOCS=Yale-Brown Obsessive Compulsive Scale; baseline MADRS=Montgomery-Åsberg Depression rating scale; § this patient (no7) was already published in a previous publication with clinical outcome [11]; * this patient suffered from obsessions in absence of compulsive behavior. Therefore, the mere obsession score (maximum 20) was counted for a surgery indication.)

No.	Sex	Age	Diagnosis	Score
YBOCS				
1	F	31	OCD	35.5
2	M	36	OCD	34.5
3	M	49	OCD	17.5^*^
4	M	50	OCD	32
5	F	52	OCD	30.5
6	M	25	OCD	35.5
*7§*	*M*	*37*	*OCD*	*40*
MADRS				
8	F	43	TRD	44

### Magnetic resonance imaging acquisition

DTI scans were acquired on a 3 Tesla scanner (Magnetom Prisma, Siemens Healthineers, Erlangen, Germany) with a 64-channel head coil and the following parameters: axial orientation, 42 slices, 1.5 × 1.5 × 3 mm^3^, TR 2800 ms, TE 88 ms, bandwidth 1778 Hz, flip angle 90°, SMS factor 3, 15 non-diffusion weighted images, 2 × 58 images with b-factors 1000 and 2000 s/mm^2^; acquisition time 6:22 min.

In addition, the following sequences were acquired:

3D-T1-weighted MPRAGE (sagittal orientation, 160 slices, 1 × 1 × 1 mm^3^, TI, 988 ms; TR, 2300 ms; TE,2.26 ms; Flip angle, 12°; GRAPPA factor 3, 3:54 min).

3D-T2-SPACE (sagittal orientation, 160 slices, 1 × 1 × 1 mm^3^; TR 2500 ms; TE 231 ms variable; 6:42 min).

#### Image fusion

After MR imaging, fusion of the individual DICOM sequences (including T2 and B0 as well as DICOM overlaid with HAMLET results) to the T1 weighted sequence is performed. For the B0 sequence with its gross distortion (especially splenium of corpus callosum and frontopolar region), we perform additional distortion correction (only for B0 to T1 fusion) with the elastic fusion algorithm (Cranial Distortion Element, Brainlab, Munich, Germany).

#### Deterministic tracking procedure

**Fig. 2:**
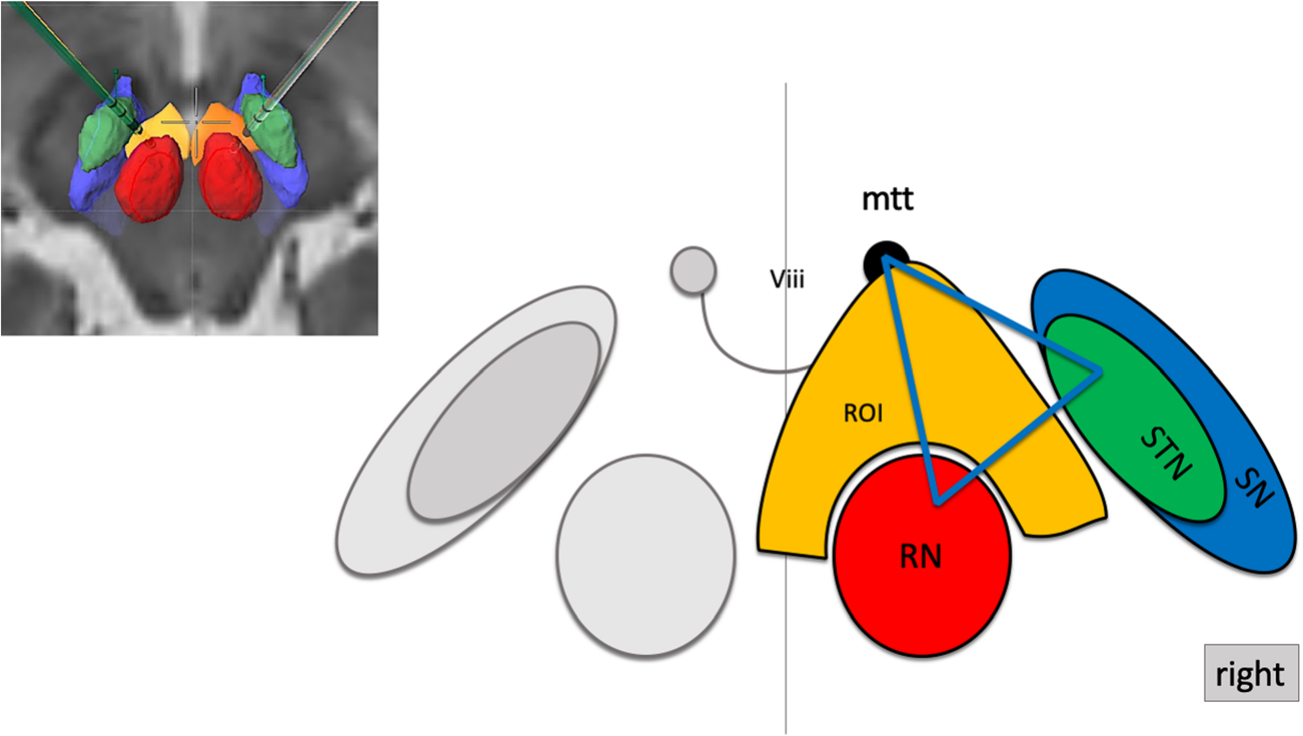
Manual definition of the ROI for deterministic fiber tracking of the slMFB. Axial slice at the level of the largest circumference of the red nucleus (RN). A single region of interest (ROI, orange; “jelly bag cap”) is drawn left and right and in front of RN in one axial slice. The ROI fills the corridor between RN and STN/SNr, crosses the midline, and includes the mammillothalamic tract (mtt). Legend: Viii, third ventricle; STN, subthalamic nucleus; SN, substantia nigra; therapeutic triangle for slMFB DBS, blue

**Fig. 3:**
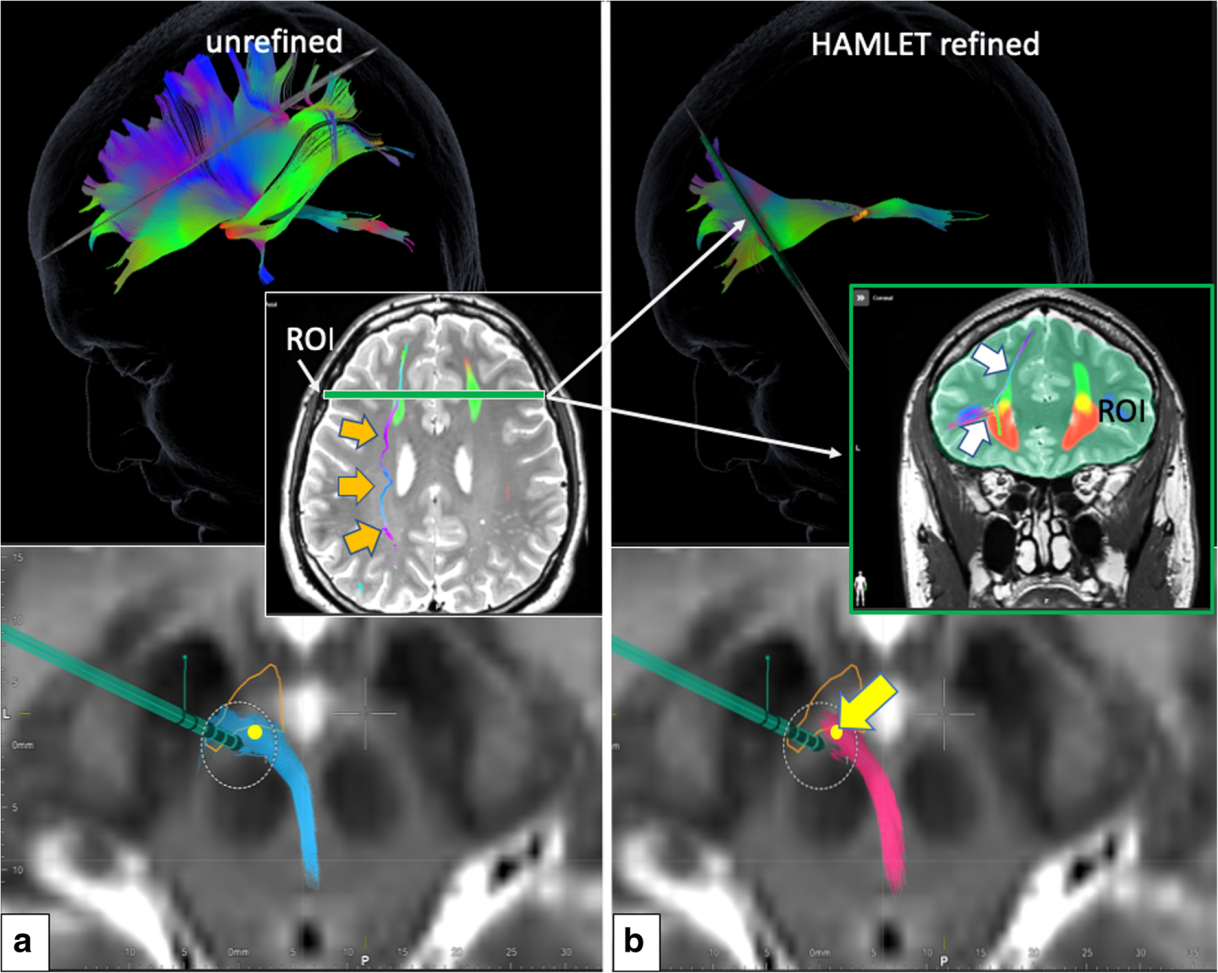
Example of deterministic fibertracking before (left panel, **a**) and after HAMLET refinement (right panel, **b**; second ROI is placed according to cortical fiber extension in HAMLET rendition). Motor fibers over-sprouted (orange arrows, left panel, axial inset) without the use of the second HAMLET-defined coronal ROI (right panel, inset). Of note (lower axial images), the simulated DBS touches the unrefined bundle (left) but is clearly misplaced if after refinement and deselection of motor fibers only prefrontal fibers are residual (yellow arrow indicates optimal DBS electrode position)

For the deterministic procedure, we used one of the two (multishell) DTI sequences with b-factor 1000. We have previously described our ROI definition for the deterministic—slMFBdet—tracking procedure [9, 10, 25] which was further adapted and refined [1]. ROI definition: minimal FA level 0.2, minimal fiber length = 80 mm, cut off angle 45–50°. We chose the axial slice (T2, ACPC—parallel) that showed the widest circumference of the red nucleus (RN). A rounded triangular shape is defined in front, lateral, and medial of the ipsilateral red nucleus, touching the midline and anteriorly including the mammillothalamic tract (“fiber tracking” element, BrainLab, Munich, Germany; Fig. 2). A second ROI (inclusion) is necessary to restrict over-sprouting of fibers and drawn around the frontal pole enclosing the orbitofrontal surface. It is useful to use the cortical and frontal posterior-most extension of the HAMLET-defined slMFBml (see below) to choose the position of this ROI (Fig. 3). The resulting streamlines of slMFBdet are then visually checked for plausibility. In the detailed anatomical definition, fibers reach BA 8, 9, 10, 11, and 11 m and some BA 47 [9, 15].

#### Machine learning–based slMFB prediction (HAMLET)

**Fig. 4:**
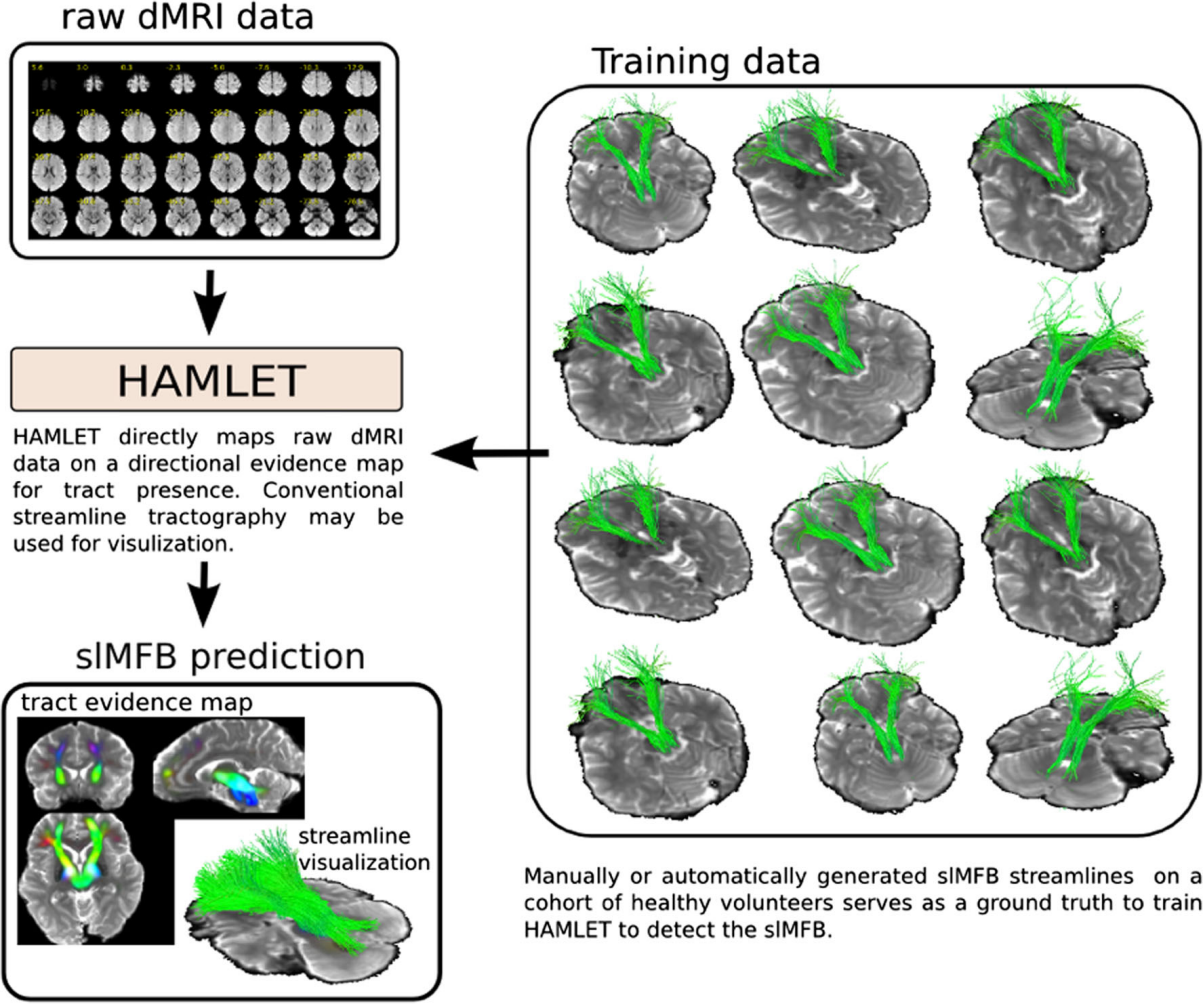
HAMLET learns from training data a direct mapping from dMRI data onto a directional tract evidence map. For each voxel of the map tells how probable it is that the sLMFB visits this voxel. The training data is automatically generated on a high-quality dataset. HAMLET can also adapt to low-quality datasets as the machine considers only low-order directional information

An extension [21] of the trainable non-linear filter described in [26] was used to learn tensor fields, such that the filter’s output can be used for classical tensor-based streamline tractography. The filter is rotation covariant, that is, the response of the filter (the tract image) always rotates according to the input (the brain). While not sounding very subtle, this property is indispensable for any tracking algorithm, however, for an ML approach not trivially to fulfill. We built such a filter to detect the superolateral branch of the medial forebrain bundle [13]. For details of algorithms, we refer to [21]; Fig. 4 shows a rough outline of the idea. The final machine—HAMLET (Harmonic hierArchy MultiscaLE Tracking)—was trained on a set of 20 healthy controls based on an automatic selection procedure (described in [13]). Only two-order dMRI information is used, i.e., any ordinary clinical protocol, which allows the estimation of a diffusion tensor, is already sufficient. The choice of the training set is rather random. Indeed, the number of training subjects was limited form a technical perspective (computer memory). However, we additionally found that the results do not heavily depend on the choice of training subjects. The number 20 was a good compromise between consumption and robustness. For visualization, it is also possible to use the prediction of HAMLET to perform ordinary streamline tracking in a sense of bundle-specific tractography [22] (Fig. 9). The idea is close to ordinary tractography, however, instead of using direct tensor data, HAMLET predictions are used (see [21] for details).

#### slMFBdet–slMFBml overlay

**Fig. 5:**
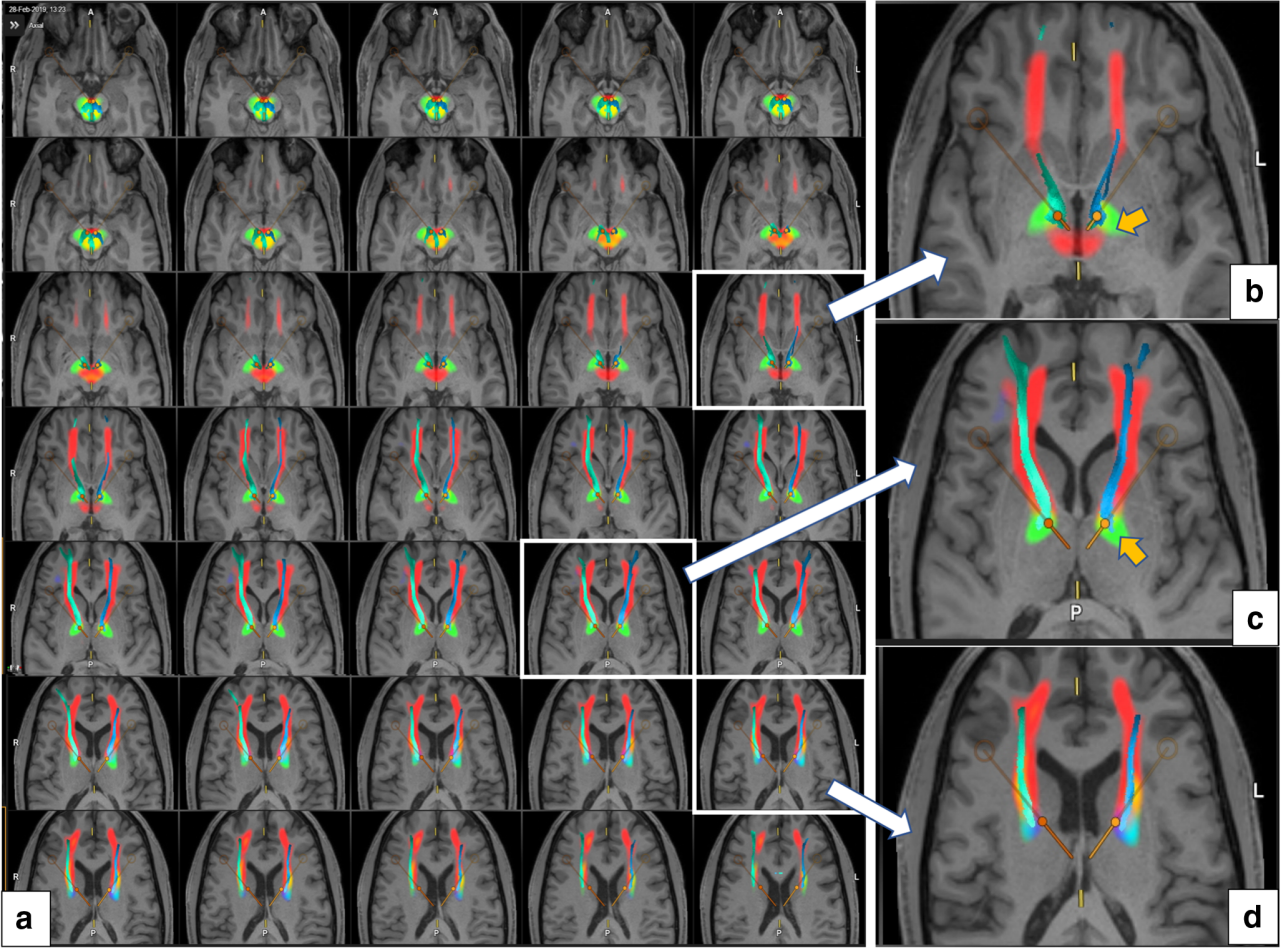
Superimposition of HAMLET slMFB rendition and slMFBdet streamlines. **a** Axial slice overview showing tract evidence maps (color-coded) and streamlines superimposed. **b**–**d** Templates magnified. Orange arrow shows fibers belonging to ansa lenticularis regularly picked up by HAMLET (green, bilaterally present)

**Fig. 6:**
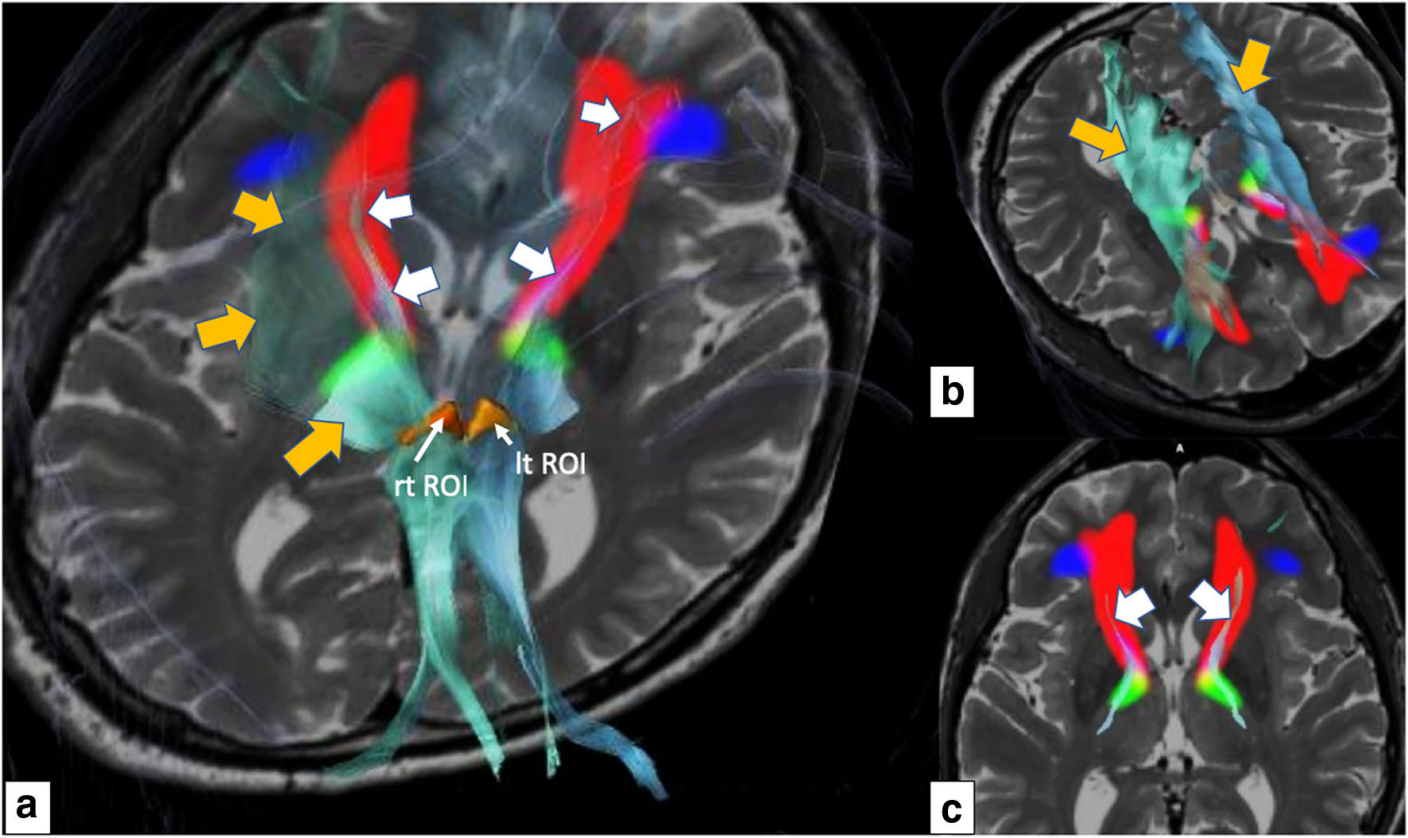
Comparison of deterministic (streamlines) and machine-learned (in-plane, colored) slMFB rendition. **a** Inferior view from right; **b**, superior view from right; **c**, axial view. OFC/PFC correct deterministic streamlines (white arrows), over-sprouting motor-related deterministic streamlines, orange arrows

After the tractographic process is visually evaluated and inspected, the anatomical T1/T2 images (DICOM) are merged with the slMFBml overlay (Figs. 5, 6). The windowing of the color coding is determined automatically (a “jet” colormap is mapped between the median of the detection map and its 90%-quantile). Values below the median are not displayed, values above the 90%-quantile onto the maximum). HAMLET was trained to show the slMFB only; thus, it omits to display motor-related fibers. If slMFBdet shows fibers that overshoot over the slMFBml visualization, the deterministic approach is revised by iteratively adjusting the ROI or adding an additional inclusion ROI as described above (frontal pole). In this respect, slMFBml serves as an objective suggestion for the deterministic tracking process. As a rule, deterministically tracked fibers should stay inside the slMFBml corridor (Figs. 5 and 6).

## Results

**Fig. 7:**
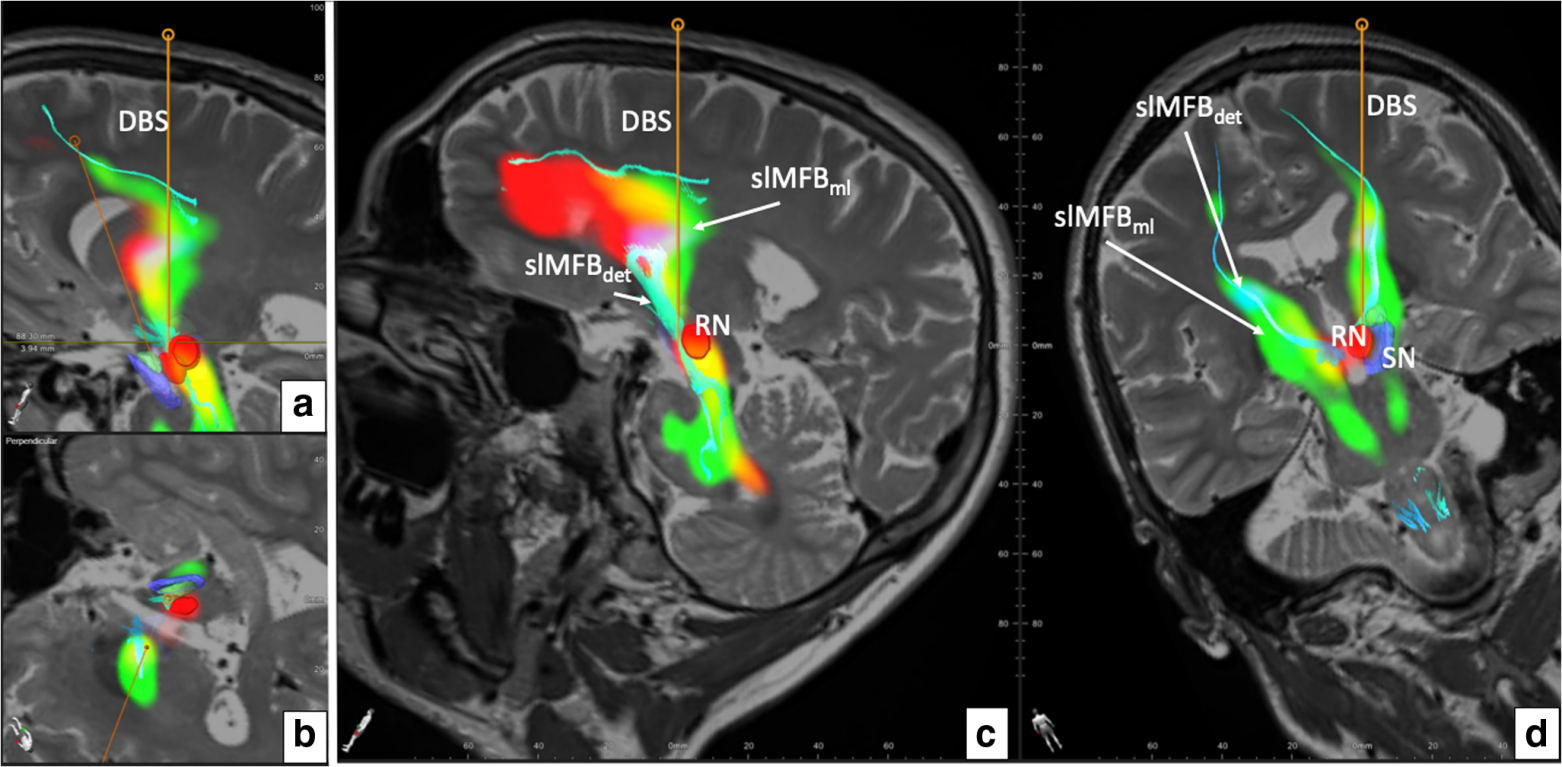
Overlay of slMFBdet and slMFBml. **a** Reconstruction along the right DBS electrode, overlay of slMFBdet, and slMFBml. **b** Cut perpendicular to right DBS electrode. Electrode does not touch nuclear environment and is placed through the slMFB center. **c** The DBS electrode is placed in the slMFB, traversing the structure in the sagittal view over the slMFB. **d** Coronal reconstruction, note how the streamlines of the slMFBdet coincide with the slMFBml. Legend: DBS, deep brain stimulation electrode; SN, substantia nigra (blue); RN, red nucleus (red)

The presented results with respect to patients are of qualitative nature and focus on the implementation process of the new heuristic in our clinical workflow. We have evaluated and tested the HAMLET approach in randomly selected *n* = 8 patients consecutively undergoing slMFB DBS for MD or OCD (an example of DBS planning is shown in Fig. 7). The detailed clinical results for a cohort including part of this here will be presented elsewhere, and we only present baseline psychometric data to characterize the population (Table 1).

### Workflow

**Fig. 8:**
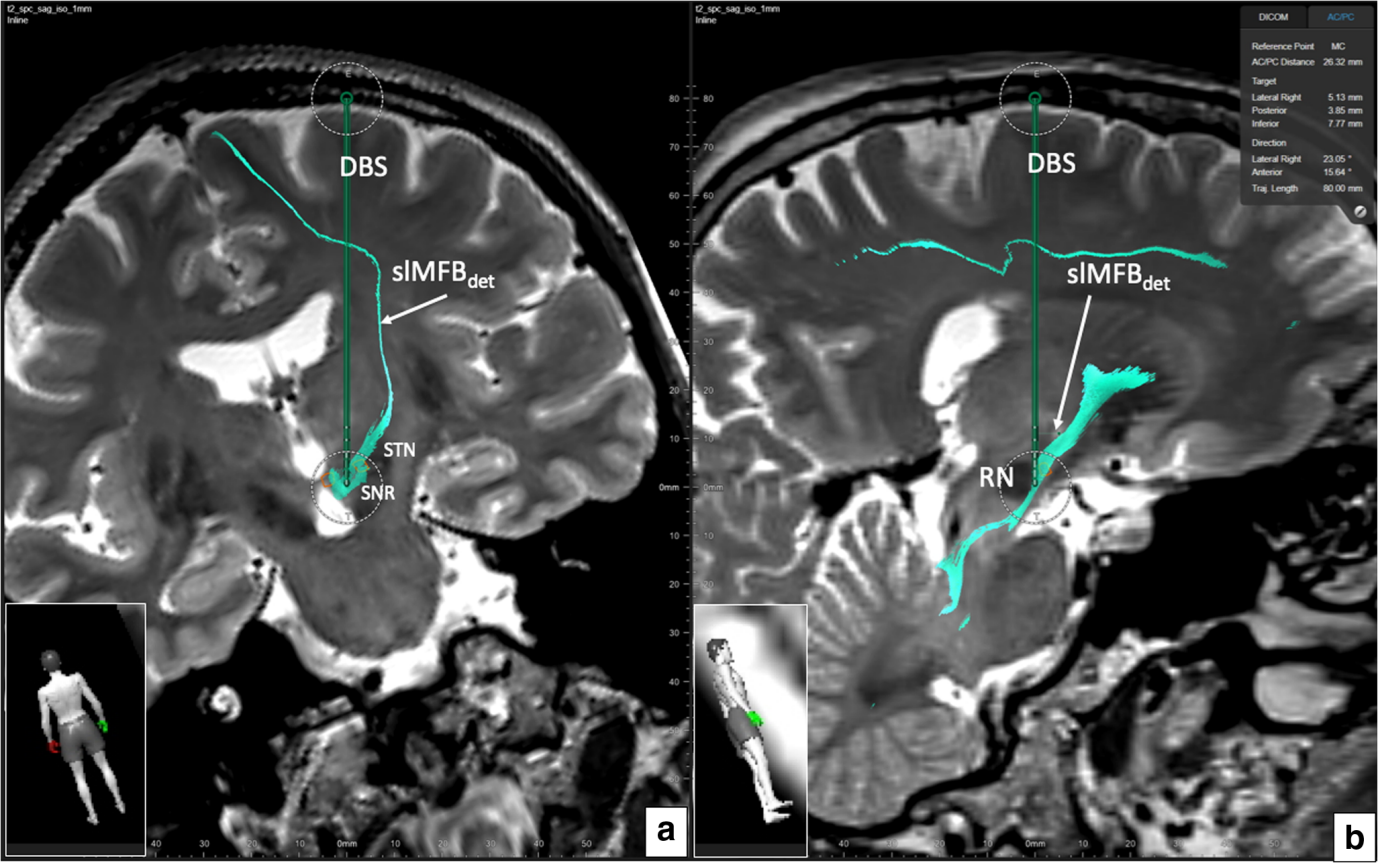
Typical bilateral slMFB rendition in 2D including the display of DBS electrode positions. Legend: DBS, deep brain stimulation electrode; STN, subthalamic nucleus; SN, substantia nigra; RN, red nucleus; slMFBdet, superolateral branch of medial forebrain bundle (in deterministic tracking)

The pipeline was implemented to work automatically. After the acquisition, DICOM sequences are send off from the scanner for HAMLET analysis (ca. 45 min), and the results are automatically stored into PACS (Picture Archiving and Communication System) together with the original data. The whole set is then transferred to the planning workstation. Automated image fusion was visually controlled and found accurate in all pairs. This holds especially true for HAMLET results overlaid DICOMs. Due to the deterministic approach and the one tensor model realized in the fiber tracking element, only some of the many anatomically described frontal lobe regions are reached. Depending on the tractographic sequence, fibers that reach the OFC (BA 11, 11 m, 47) are typically not visualized, and the depicted streamlines sometimes shear out medially or laterally as an alternative. Because of its main direction, streamlines reach rostral parts of the superior frontal gyrus (BA8, 9, 10). Also, motor-related over-sprouting fibers (reaching BA 4, 6) are displayed when only using the first ROI. These fibers are eliminated by using a slMFBml determined second ROI (Fig. 5b). In our series, no further refinement steps were necessary. As to be expected, HAMLET in all cases showed the slMFB in its extensions as previously described [1, 9, 13]. In the deep-seated white matter, HAMLET is not able to exclude motor fibers related to ansa lenticularis (green in Fig. 6, orange arrows) which run lateral to the slMFB, a phenomenon only appearing in very deep-seated regions of the midbrain. This has not been a problem, and erroneous fibers in this region were in no case captured by deterministic tracking. HAMLET did not show details of the midbrain target region (Figs. 4 and 5) and surgical targeting always was performed on deterministic streamlines (Fig. 8).

**Fig. 9:**
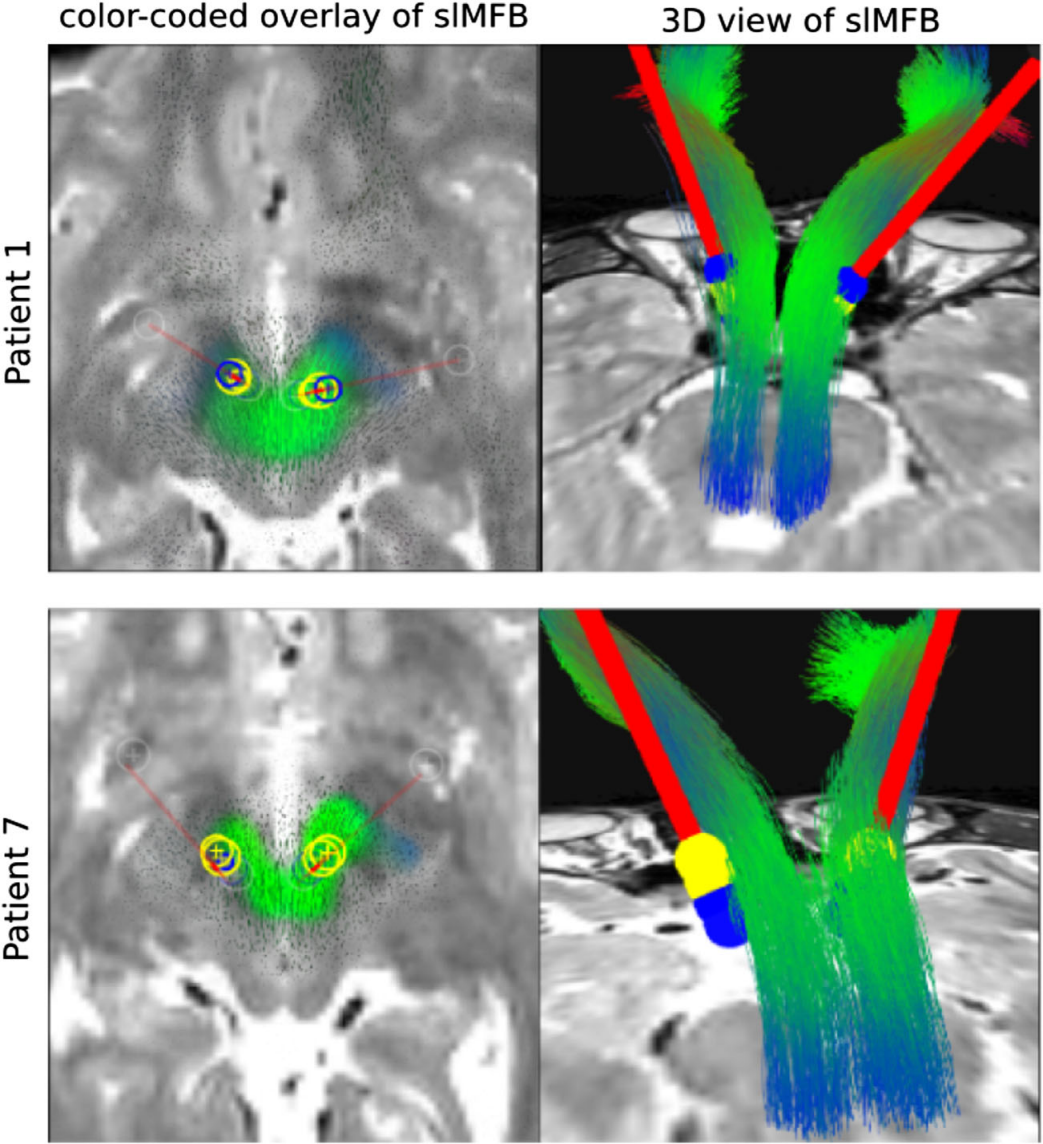
Electrode position for two cases relative to HAMLET’s slMFB predictions for two cases. For a depiction of the slMFB, a T2 color-coded overlay is used (left) and additionally a bundle-specific tractography (right). For patient 1, the electrodes are well placed; for patient 7, the left electrode is sub optimally located (lateral and posterior shift). Clinically, patient 7 showed no optimal improvement to DBS

In an exemplary analysis, we investigated two bundle-specific tractographies of slMFB relative to electrode positions (Fig. 9). For patient 1, both electrodes appear to be well placed and she responded well with here YBOCS score. However, confluent with lesser than expected YBOCS improvement in patient 7, the left electrode to HAMLET’s prediction is located too lateral and posterior.

## Discussion

We have here introduced and tested a combined machine learning approach based on individual DTI sequences that automatically displays slMFB anatomy acutely after MRI acquisition. In the presented cases, deterministic streamlines (slMFBdet) in all cases stayed inside the corridor given by HAMLET (slMFBml) once over-sprouting motor fibers (cortical) were excluded by the introduction of a second ROI (frontal pole). Nevertheless, the slMFBml (HAMLET) heuristic serves as an important safety to check for optimal fiber selection.

The ROI definition was adapted from our earlier results including suggestions from Anthofer et al. [1] who found advantages in using part of the dorsal raphe nucleus (DRN) as a midline structure for ROI definition. This makes sense since the VTA is in its most dorsal part (caudal linear nucleus) intimately connected to the DRN [19]. The rounded triangular ROI (Fig. 2) realizes this idea, and only the fibers connecting VTA toward OFC and PFC must be included in the “surgical display” of the slMFB [13]. In order to exclude motor-related fibers, sometimes ROI must be modified, typically reducing its lateral extension. Another strategy is to include a second frontal ROI that encloses the frontal pole and the OFC and is positioned according to the HAMLET fiber display (Fig. 3, right). In addition to the elimination of over-sprouting motor-related fibers in the process of deterministic tracking, the identification of the bundle itself can be difficult. Topographic anatomical knowledge about regions of the PFC and OFC is needed for a visual appreciation [1, 13], and even more detailed knowledge about Brodmann regions [6] would be helpful. In this respect, a detailed and patient-specific map available for individual planning of the slMFB like presented here might simplify the surgical approach. One way to achieve this goal would be by utilizing a normative connectivity map. Other groups use these normative maps in conjunction with patient-specific imaging in order to show targeted fiber systems [18, 23, 24]. By this, they enhance patient-specific anatomy, while assuming that normative connectomic maps can serve as a blueprint to refine deterministic tractography and plan electrode positions and efficacious treatment. We here offer another solution: The information used to delineate the slMFB in both scenarios is based on the same patient-specific DTI sequence. After a detailed tracking procedure employing a deterministic approach, the results are checked and refined in the patient-specific surgical planning environment by direct overlay with slMFB anatomy derived by a machine learning approach (HAMLET; Figs. 4, 5, and 6). This heuristic HAMLET merely serves as a road map or tracking corridor that helps to appreciate and to refine the results of the deterministic (surgical) targeting procedure.

### Limitations

There are certain limitations of the planning process that are mostly related to the deterministic tracking approach. Deterministic tracking (typically in a one tensor approach) is part of most surgical planning systems (with CE mark or FDA approval) and regarded as robust. The approach, however, shows certain problems with resolving kissing, crossing, and sprouting fiber situations [7, 8, 22]. It is thus to be expected that the algorithm will under-represent streamlines of a target structure with such complex branching into PFC/OFC segments like the slMFB [1, 13]. However, in the slMFB target region directly, this distant underrepresentation does not play a major role [13]. A bigger issue appears to be an over-sprouting of motor-related fibers which are directly connecting the VTA and the motor-related cortical regions (BA4, 6) [1]. The significance of these fibers is yet to be cleared up. These fibers can alter the target region (Figs. 3 and 6) but can reliably be excluded with the simple introduction of a second inclusion ROI (this is the concept of normal tractography). HAMLET appears to occasionally show motor fibers but only in very deep-seated regions of the midbrain (typically ansa lenticularis, Figs. 5b and 6). Here, the algorithm can overestimate the slMFB laterally. Moreover, HAMLET does not show details of the target region (Fig. 5a) but merely a broad corridor. This corridor is of value since it is displayed based on individual imaging and in our eyes helps to appreciate deterministic slMFB anatomy and only in combination with slMFBdet rendition.

HAMLET is almost immediate (45-min calculation time) and has the advantage of helping to define an objective corridor for the deterministic streamline tractography at the day of MRI acquisition. To a certain extent, the described process can be referred to as self-referential, and the same data is analyzed twice: once with deterministic tractography and then with and compared to HAMLET which uses the same individual DTI data set and computes the slMFB based on a training cohort that was tracked according to our idea of correct slMFB rendition [21]. HAMLET allows an objective individual definition of the slMFB with deterministic tractography which serves as the surgical target. HAMLET thus does not necessarily show anatomical ground truth but merely *an improved retest reliability*.

Human slMFB anatomy has been described based on DTI due to a lack of anatomical ground truth [9, 12, 13]. This description was subsequently used to teach HAMLET slMFB anatomy. While we ourselves are convinced of the anatomical, display, and the associated physiological function of the structure, there is a minor possibility of a subsequent fault in tract rendition and in this instance, we would reliably repeat the display of a non-existent or ill-defined tract.

By definition of an anatomical “ground truth” for the slMFB, which is then used during training, a certain reference knowledge is introduced, which is of course to a certain extent subjective. However, at this moment, a better ground truth beyond previous publications based on DTI is not available.

However, in the light of the very promising DBS results in MD [2, 14, 25] and OCD [11, 17]—which are based on tractographic DBS applied to slMFB anatomy—we think that this is very unlikely.

### Outlook

In the future, we can envision expert systems which might be directly integrated into surgical planning systems (“anatomically constrained tracking” [22, 27]) and will allow personalized semi-automatic or even fully automatic surgical display of targeted fiber tracts using machine learning algorithms to guide advanced tracking solutions. Moreover, the availability of internet-based cloud systems will help to compare target structures between institutions and add to the inter-expert data exchanges. Moreover, effective electrode positions together with the effectively stimulated volume of tissue activation studies (in normative brain rendition) can be exchanged serving as a reference for individual planning. The IT technology is already available, but patient confidentiality and data safety are important issues yet to be solved especially in the international exchange of larger data [16].

## Conclusion

The slMFB is a complex fiber structure which on the individual level cannot be entirely depicted with deterministic tractography approaches. The main slMFB midbrain target region can be readily identified based on the deterministic approach, given that over-sprouting of fibers not directly belonging to the slMFB is prevented. We have here introduced a concept for an individual and objective tractographic planning approach which combines a machine learning approach (HAMLET) with patient-specific deterministic tractography and surgical targeting. The defined pipeline allows the comparison of both modalities in real time in a commercial planning environment. This approach is not intended to present “anatomical ground truth” but rather to perform deterministic tractography to reliably define a surgical target. This approach might in the future help to reliably perform slMFB DBS making it available for less-experienced groups while at the same time helping to reliably deliver an appropriate stimulation efficacy. The here described strategy can in principle be applied for any tractographically derived target structure.
